# The self-care behaviors and health literacy can play important preventive roles in older female osteoporosis

**DOI:** 10.1186/s12905-023-02546-2

**Published:** 2023-08-11

**Authors:** Elahe Malekmirzaei, Azizeh Farshbaf-Khalili, Vahid Pakpour

**Affiliations:** 1 School of Nursing and Midwifery, Student Research Committee, Tabriz University of Medical Sciences, Tabriz, Iran; 2 Physical Medicine and Rehabilitation Research Center, Aging Research Institute, Tabriz University of Medical Science, Tabriz, Iran; 3 Department of Community Health Nursing, School of Nursing and Midwifery, Department of Geriatric Health, School of Health, Tabriz University of Medical Sciences, Tabriz, Iran

**Keywords:** Osteoporosis, Elderly, Self-care, Health literacy, Prevention

## Abstract

**Background:**

Osteoporosis is a common complication of aging and menopause. Self-care and health literacy are among the factors affecting health status. The purpose of this research was to determine the preventive roles of self-care behaviors and health literacy in older women with osteoporosis.

**Methods:**

This cross‑sectional analytical research was conducted on 250 postmenopausal women consisting of 125 osteoporotic and 125 healthy people aged 60–70. They were selected by purposive sampling in Tabriz Sina Hospital from September 2021 to December 2021. Data collection instruments were a demographic questionnaire, a Menopausal Self‑Care Questionnaire, and a European Health Literacy Survey Questionnaire. Data were analyzed using SPSS 23 software.

**Results:**

The mean (SD) total score of self-care in healthy women was 118.97 (19.92) and in women with osteoporosis was 84.7 (14.98) (p < 0.001). Also, healthy women all had sufficient health literacy (100%), but 52.8% of women with osteoporosis had insufficient health literacy. The odds of osteoporosis decreased significantly with the rise in the total score of self-care behaviors [Odds ratio 95% confidence interval (95% CI); p: 0.909 (0.880 to 0.939); p < 0.001] and its subdomains as well as with increasing health literacy level [OR (95% CI); p: 0.322 (0.266 to 0.383); p < 0.001]. There was a significant positive correlation between self-care behaviors and health literacy (r = 0.616, p < 0.001).

**Conclusions:**

Self-care in aged women is particularly important in reducing the risk of osteoporosis, and empowering women in the field of health literacy is an important factor in improving self-care behaviors and ultimately the health of these people.

**Supplementary Information:**

The online version contains supplementary material available at 10.1186/s12905-023-02546-2.

## Introduction

Osteoporosis is defined as a systemic skeletal disease that involves gradual and silent bone loss. It is characterized by deterioration of bone tissue microarchitecture, increased bone fragility, and bone susceptibility to fracture, which leads to reduced dynamic balance in patients [1]. Based on the results of a large population-based study in Iran, the age-standardized prevalence of osteoporosis was 24.6% in men and 62.7% in women over 60 years of age [2]. The result of a review study in Europe shows that women are four times more at risk of osteoporosis compared to men [3]. Therefore, it can be stated that postmenopausal women are at a high risk of osteoporosis and its complications [4]. The reason for the difference between men and women in this regard can be attributed mainly to hormonal changes after menopause [5].

Osteoporosis is often attributed to various factors such as environmental factors, genetics, and lifestyle. According to previous studies, the insufficient intake of calcium and vitamin D, the lack of proper physical activities, and unhealthy habits and behaviors such as smoking and alcohol consumption are the known causes of osteoporosis [6]. Therefore, lifestyle modification with an emphasis on bone health habits is recommended for healthy postmenopausal women [7].

Since prevention of behavioral habits affecting osteoporosis requires a change in the individual’s behavior, self-care behaviors can play an important role in preventing many older women from developing this disease. Self-care behaviors are primarily advised to healthy people, and their aim is the prevention of diseases. However, these behaviors are also necessary for the health of people with chronic diseases [8, 9]. Self-care is defined as a strategy to adapt to the events and tensions of life, which will lead to independence, and it includes certain activities by which the symptoms of the disease are alleviated and the health of the patient is maintained [10]. People with osteoporosis do not always have sufficient confidence and ability to adopt healthy behaviors and prevent osteoporosis and thus, they face real challenges in controlling and managing their disease [11].

The use of self-care practices has led to favorable outcomes such as reduced complications, improved feeling of control, responsibility, independence and reduced dependence on others, improved adaptation, and promoted the feeling of well-being [10]. Previous studies have dealt with factors such as self-efficacy, age, gender, and educational attainment as influential components in self-care behaviors [12, 13]. Among these, health literacy has been introduced as one of the important components in self-care [8].

Health literacy is broadly defined as the ability of people to access health information and use it to make appropriate decisions about maintaining and improving their health [9, 14, 15]. Health literacy is an important element in a woman’s ability to face health promotion and prevention activities [16]. The results of various studies have shown that low levels of health literacy have significant effects on patients’ behavior and health outcomes [17, 18]. It can be argued that people with a higher literacy level are probably more aware of self-care methods such as screening tests, stress management, etc., and these factors have led these people to have better self-care compared to other people. According to Javadzadeh et al., the majority of the referrals of people with high health literacy levels are due to a variety of check-ups and prevention measures, not the illness itself [19].

Based on the results of studies conducted in this line of inquiry, there is an urgent and dire need to improve the level of health literacy and highlight the importance of self-care in chronic diseases including osteoporosis. Given the alarming scarcity of studies on health literacy and self-care in older osteoporotic women and their healthy counterparts in Iran, and the contradictory results of such studies conducted in other countries, the present study was conducted to determine the preventive role of self-care behaviors and health literacy in older women with osteoporosis as main objective as well as the relationship between self-care behaviors and the level of health literacy in older women with osteoporosis versus older healthy women. The aim was to identify the important factors playing a role in the timely prevention and diagnosis of osteoporosis and to design programs to reduce the incidence of this disease among women.

## Materials and methods

### Study design and participants

This research is based on STROBE statement. The present study was a cross-sectional, descriptive- analytical study conducted in Tabriz, Iran, in between October 1400 and January 1400. Tabriz, the largest city in the northwest of Iran, is the capital of East Azerbaijan province. Tabriz is the third largest city in Iran (in terms of area). The population of Tabriz was 1,584,855 people in 2016 (City proper and suburbs: 1,727,476 people) and reached 1,643,960 people in 2022. The female population of Tabriz is 799,254 people, of whom 163,228 (20.4%) are in the age group of 50 years and older. This study was confirmed by the regional ethics committee (IR.TBZMED.REC.1400.343). The statistical population of this research included elderly postmenopausal women who were referred to the bone density measurement center of Sina Hospital in the central core of the Tabriz metropolis. The participants were selected among females referring to the center who were divided into two groups of women with osteoporosis and healthy women through purposive sampling. Finally, the eligible women were selected and were then given a brief explanation of the objectives and method of the research, and upon their willingness to participate in the study, they were asked to answer the questions of the questionnaire accurately. During data collection, the participants were briefed on the objectives of the research, voluntary participation in the study, and the confidentiality of their information. After written informed consent was obtained from them, the questionnaires were completed by the researcher by interviewing the elderly women.

Elderly postmenopausal women were eligible to participate in the study if they: were aged 60 and older, had a lumbar spine or pelvic T-score equal to or greater than − 1 (for healthy women) and equal to or less than − 2.5 (for women with osteoporosis), were able to communicate verbally to answer questions, and were willing to participate in the study. Exclusion criteria were: suffering from bone diseases other than osteoporosis, metastatic bone diseases, malignancies, kidney failure and kidney diseases, taking drugs that affect bone metabolism including intravenous bisphosphonates in the last 5 years, taking oral bisphosphonates in the last 6 months, cumulative use of oral bisphosphonates for more than three years or more than one month between 6 and 12 months prior to the study, taking parathyroid hormone analogs during the last 12 months, taking hormonal drugs or corticosteroids, hereditary diseases (hemophilia, thalassemia, hemochromatosis), endocrine diseases (Cushing’s syndrome, hyperthyroidism, type 1 diabetes, primary hyperthyroidism), chronic liver diseases, abnormalities of the biliary system and disorders in the digestive system (chronic liver diseases such as primary biliary cirrhosis, celiac disease, Crohn’s disease, and partial or total gastrectomy), body mass index less than 18.5, smoking, alcohol and drug use, and unwillingness to continue participation.

### Data collection

Bone mineral density (BMD) was examined by dual‑energy X‑ray absorptiometry (DEXA) in the total hip and lumbar spine area using Hologic QDR 4500 W (S/N 50,266) dual-energy X‑ray absorptiometry device. The data collection tool in this study included three questionnaires. A demographic questionnaire was used to collect information about age, age of menopause, educational attainment, type and amount of physical activity, parity, history of smoking, history of abortion, history of fracture due to osteoporosis, and taking calcium and vitamin D supplements.

The second questionnaire was the Menopausal Self-Care Questionnaire (MSCQ), which is used to examine self-care in menopausal women. This questionnaire was designed by Kafaei Atrian et al. according to Waltz’s method for instrument development based on the conceptual framework of Orem’s self-care theory. It has 33 items in four dimensions: physical, mental, social, and information acquisition. This questionnaire is scored based on a 5-item Likert scale (never, rarely, sometimes, often, and always). The range of scores for each domain is as follows: general health care (9–45), screening [6–30], nutrition [5–25], memory [3–15], hot flashes and night sweats [3–15], sexuality [3–15], and social communication [4–20]. The total score is ranged between 33 and 165. This questionnaire was psychometrically evaluated in Iran by Kafaei Atrian et al. They reported a content validity ratio of 0.7, a content validity index of 0.7, an ICC rate of 0.76, and a Cronbach’s alpha of 0.88 [30].

The third questionnaire was the European Health Literacy Survey Questionnaire (HLS-EU-Q16) which is used to check the level of health literacy. This questionnaire examines factors such as access to and understanding of information related to health care, diseases, prevention, and health promotion. It is organized into three health domains: health care, disease prevention, and health promotion [31]. This questionnaire has 16 questions that are scored based on a 5-point Likert scale (relatively easy, easy, relatively difficult, difficult, and I don’t know). The scores range from 0 to 16. Health literacy scores are divided into three categories: 0–8: “inadequate”, 9–12: “problematic”, and 13–16: “sufficient”. The Newest Vital Sign (NVS) test was used to check the concurrent validity of this questionnaire [32]. Cronbach’s alpha obtained for HLS-EU-Q16 was reported to be 0.79 [31].

In this study, the forward-backward method was used to translate HLS-EU-Q16. The original version was first translated separately by two independent Farsi-speaking individuals fluent in English and one expert in the field of medicine. The three translators then produced an agreed version. This Persian version was translated into English by a fourth translator who had not read the original version and was not involved in the translation process of the original version. Finally, the two versions of the translation and re-translation were compared with the original version, and the final agreed Persian version was obtained. This questionnaire was given to 20 eligible women to check the accuracy and comprehensibility of the questions. Then, to confirm its validity, face validity was evaluated, and to determine the reliability of the instrument, two methods of internal consistency and test-retest were used. Face validity evaluation involved examining the opinions of the research supervisor and 10 professors of Tabriz University of Medical Sciences. As for reliability, a Cronbach’s alpha of 0.792 was obtained, and the intraclass correlation coefficient was 0. 946 (0.95% CI, p): 0.946 (0.979 to 0.864, < 0.001).

### Statistical analysis

SPSS version 23 (IBM SPSS Statistics, IBM Corporation, Chicago, IL) was used for data analysis. First, the normality of data distribution in each group was checked. Data was described using mean (standard deviation: SD) or number (percent). Chi‑square, Fisher’s exact test, independent t‑test, and Mann–Whitney U were used for the data analysis. For univariate analysis of the relationship between self-care behaviors and the level of health literacy, Pearson’s correlation test was used. A binary logistic regression model adjusted for confounding variables (age, menopausal age, education level, income, BMI, exercise, supplement use, fracture history, and exposure to direct sunlight), was used for estimating the odds ratio of osteoporosis. The Hosmer–Lemeshow test was applied for the goodness of fit for the logistic regression model. The p-values less than 0.05 were considered statistically significant.

## Results

In this study, 548 women aged 60 to 70 were evaluated in terms of eligibility criteria. Among them, 53 women were excluded from the study due to their unwillingness to participate in this research, and 245 women due to their ineligibility. Finally, 125 eligible healthy women and 125 women with osteoporosis were included in the study and the necessary information was collected.

According to the results of the demographic characteristics of aged women participating in this study, these women were in two age groups 60–65 years (26%) and 66–70 years (74%). Most of them were married (91.6%) and housewives (97.2%). In terms of economic status, about half of the women in both groups reported that they only managed to make ends meet. Statistically significant differences were observed between the two groups of with osteoporosis and healthy women in terms of mean age (p < 0.001), menopausal age (p < 0.001), exercising history (p = 0.001), exposure to direct sunlight (p < 0.001), and the type of consumed supplement (p = 0.001). Complete details are reported in Table 1.

**Table 1:** Comparison of demographic characteristics of postmenopausal women in two osteoporotic and healthy groups (250 people)

Variable	Osteoporotic group (n = 125) n (%)	Healthy group (n = 125) n (%)	Total	p-value
Age (years) †	62.30 (2.4)	64.59 (2.6)		< 0.001^*^
60–65	97 (77.6%)	60 (48.00%)	157 (62.8%)	
66–70	28 (22.4%)	65 (52.00%)	93 (37.2%)	< 0.001 ƍ
Menopausal age (years)†	49.30 (4.10)	49.58 (4.19)		0.594^*^
Marital status				1.0 ƍ
Single, widowed, divorced	10 (8.0%)	11 (8/8%)	21 (8.4%)	
Married	115 (92.0%)	114 (91.2%)	229 (91.6%)	
Job				0.466 ƍ
Housewife	120 (96.0%)	123 (98.4%)	243 (97.2%)	
Employed	5 (4.03%)	2 (1.6%)	7 (2.8%)	
Income level				0.301ǂ
Enough	27 (21.6%)	19 (15.2%)	46 (18.4%)	
Income equals expenditure	65 (52.0%)	(51.2%)64	129 (51.6%)	
Expenditure > income	33 (26.4%)	42 (33.6%)	75 (30.0%)	
Housing situation				0.759^¥^
Personal	60 (48.0%)	62 (49.6%)	122 (48.8%)	
Rental	48 (38.4%)	43 (34.4%)	(36.4%)91	
Relative’s house	17 (13.6%)	20 (16.0%)	37 (14.8%)	
Level of education				0.317ǂ
Illiterate	4 (3.2%)	6 (4.8%)	10 (4%)	
Under diploma	111 (88.8%)	115 (92.0%)	226 (90.4%)	
Diploma & university	10 (8.0%)	4 (3.2%)	14 (5.6%)	
Exercising history				0.001 ƍ
Yes	0	67 (53.6%)	67 (73.2%)	
No	125 (100%)	58 (46.4%)	183 (73.2%)	
History of fracture				0.132 ƍ
Yes	16 (12.8%)	8 (6.4%)	24 (9.6%)	
No	109 (87.2%)	117 (93.6%)	226 (90.4%)	
Family history of fractures due to osteoporosis				0.220 ƍ
Yes	12 (9.6%)	6 (4.8%)	18 (7.2%)	
No	113 (90.4%)	119 (95.2%)	232 (92.8%)	
Being exposed to direct sunlight	45 (36.0%)	79 (63.2%)	124 (49.6%)	< 0.001^¥^
Supplement use				0.639 ƍ
Every day regularly	11 (8/8%)	0	11 (8/8%)	
Every other day	12 (9.6%)	0	12 (9.6%)	
One per week	102 (81.6%)	4 (100%)	106 (82.2%)	
Consumed supplement				0.001^¥^
Vitamin D	76 (60.8%)	0	76 (30.4%)	
Calcium	10 (8.0%)	2 (1.6%)	12 (4.8%)	
Vitamin D & calcium	38 (30.4%)	1 (0.8%)	39 (15.6%)	
Soy supplement	1 (0.8%)	21 (0.8%)	22 (0.8%)	
No supplementation	0	121 (96.8%)	121 (48.4%)	
BMI (kg/m^2^)†	23.4 (3.23)	24.1 (3.49)	-	0.790^*^

All numbers are reported as counts (percentages) except those marked with †, which represent the mean (standard deviation). *Independent t-test ǂ Trend Chi-square ¥ Chi-square ƍ Fisher’s exact test

BMI: Body mass index

The mean (SD) of the total score of self-care in healthy and osteoporotic women was 118.97 (14.98) and 84.7. (19.92), respectively, and this difference was statistically significant (p < 0.001). Also, the mean scores in all self-care domains including general health care (p < 0.001), screening (p < 0.001), nutrition (p < 0.001), memory (p < 0.001), hot flashes and sweating (p < 0.001), sexuality (p < 0.001), and social communication (p < 0.001) were significantly higher in healthy women compared with those suffering from osteoporosis (Table 2).

**Table 2:** Comparison of self-care behavior in two groups of osteoporotic and healthy older women

Self-care subdomains (score domain)	Osteoporotic group (n = 125) mean (SD)	Healthy group (n = 125) mean (SD)	t	df	*p-value
General health care (9–45)	21.11 (6.55)	33.26 (5.85)	45.15	248	< 0.001
Screening (6–30)	12.00 (4.48)	14.22 (5.62)	3.45	236.23	< 0.001
Nutrition (5–25)	14.85 (4.82)	21.69 (3.35)	13.01	221.33	< 0.001
Memory (3–15)	6.30 (5.4)	9.13 (2.46)	13.01	221.33	< 0.001
Hot flashes and sweating (3–15)	10.52 (3.24)	13.57 (1.88)	9.07	199.41	< 0.001
Sexuality (3–15)	6.30 (2.14)	9.13 (2.46)	9.69	243.18	< 0.001
Social communication (4–20)	13.67 (4.54)	17.94 (2.81)	8.93	207.10	< 0.001
Total score (33–165)	84.7(19.92)	118.97 (14.98)	15.33	230.27	< 0.001

* Independent t-test

A comparison of health literacy levels in the two study groups showed that all healthy women had sufficient health literacy (100%), while 52.8% of women with osteoporosis had insufficient health literacy. Statistical analysis showed a statistically significant difference between the two groups in terms of their health literacy levels (Table 3).

**Table 3:** Status of health literacy in two groups of osteoporotic and healthy older women

Health literacy score and its levels	Osteoporotic group (n = 125) mean (SD)	healthy group (n = 125) mean (SD)	*P-value
Health literacy (0–16)	7.76(3.58)	14.64 (0.98)	< 0.001*
Insufficient (0–8)	n (%) 66 (52.8%)	n (%) 0	
Problematic (9–12)	55 (44.0%)	0	< 0.001^±^
Enough (13–16)	(3.2%)4	125(100%)	

* Independent t-test ± Mann-Whitney U test

Table 4 shows the results of the multivariate logistic regression model used to compare the scores of self-care and its domains in postmenopausal women with and without osteoporosis. This model was adjusted for age, menopausal age, Body Mass Index (BMI), educational attainment, income, exercise, supplement use, fracture history, and exposure to direct sunlight. The odds of having osteoporosis decreased significantly with the rise in the total score of self-care behaviors [Odds ratio (OR): 95% confidence interval (95% CI); p: 0.909 (0.880 to 0.939); p < 0.001] and its subdomains and with increasing health literacy level [OR (95% CI); p: 0.322 (0.266 to 0.385); p < 0.001].

**Table 4:** The results of the multivariate logistic regression model to estimate the odds ratio of osteoporosis based on self-care behavior and health literacy

Self-care behaviors & Health literacy	Adjusted odds ratio (95% confidence interval))	p-value^€^
Self-care behaviors General Health care	0.806 (0.748 to 0.869)	< 0.001
Screening	0.908 (0.831 to 0.985)	0.019
Nutrition	0.762 (0.685 to 0.8471)	< 0.001
Memory	0.655 (0.531 to 0.808)	< 0.001
Hot flashes and sweating	0.730 (0.616 to 0.866)	< 0.001
Sexuality	0.655 (0.531 to 0.808)	< 0.001
Social communication	0.799 (0.710 to 0.899)	< 0.001
Total score	0.909 (0.880 to 0.939)	< 0.001
**Health literacy**	0.322 (0.266 to 0.385)	< 0.001

€ Logistic regression test adjusted for age, menopausal age, education level, income, BMI, exercise, supplement use fracture history, and exposure to direct sunlight

Regarding the correlation between self-care and health literacy in older women, our findings indicated that in all women participating in the study, the total score of self-care behavior and all its domains had a statistically significant and positive correlation with the level of health literacy (p < 0.001). However, analysis of this correlation by groups (i.e., osteoporotic vs. healthy women) showed a significant positive correlation between the total score of self-care behavior and all its domains with the level of health literacy in the osteoporosis group (r = 0.616, p < 0.001), but no such correlation was observed in healthy women (Table 5).

**Table 5:** The relationship between self-care behaviors and the level of health literacy in two groups of sick and healthy older women

Subdomains of self-care behaviors (score range)	Health literacy level					
	All women		Osteoporotic		Healthy	
	r ^€^	P *	r ^€^	P *	r ^€^	P *
General health care	0.539	< 0.001	0.352	< 0.001	-0.006	0.949
Screening	0.337	< 0.001	0.297	< 0.001	0.154	0.086
Nutrition	0.557	< 0.001	0.361	< 0.001	-0.039	0.664
Memory	0.414	< 0.001	0.213	< 0.001	0.070	0.441
Hot flashes and sweating	0.559	< 0.001	0.479	< 0.001	-0.103	0.254
Sexuality	0.414	< 0.001	0.213	< 0.001	0.070	0.441
Social communication	0.533	< 0.001	0.451	< 0.001	-0.101	0.261
Total self-care	0.616	< 0.001	0.493	< 0.001	-0.068	0.451

* Pearson ^€^ Correlation coefficient

## Discussion

The present study was conducted to investigate the preventive role of self-care behaviors and health literacy in older women osteoporosis as main objective as well as to examine the relationship between self-care behaviors and health literacy among healthy and osteoporotic postmenopausal older women who referred to the bone densitometry center of Sina Hospital in Tabriz, Iran. According to the results, there was a significant difference between the two groups in terms of their age, menopausal age, exposure to sunlight, exercising history, and consumption of supplements. Also, self-care behaviors and all its domains (including general health care, screening, nutrition, memory, hot flashes and sweating, sexuality, and social communication) had a significant relationship with osteoporosis. A similar relationship was also observed between the level of health literacy and osteoporosis. It was also observed that for every one-unit increase in the scores of general health care, screening, nutrition, memory, hot flashes and sweating, sexuality, and social communication, the odds of developing osteoporosis fell by nearly 20%, 10%, 25%, 35%, 25%, 35%, and 20%, respectively. By the same token, for every one-unit increase in the total score of self-care, the odds of developing osteoporosis decreased by 10%. In addition, for every one-unit increase in the total score of health literacy, the odds of getting osteoporosis were reported to be 0.322%. There was a significant positive relationship between self-care behaviors and the level of health literacy in all osteoporotic and healthy women together, and in osteoporotic women alone.

Keramat et al. [22] stressed the effect of environmental factors such as nutrition, exercise, and exposure to sunlight on osteoporosis, which is in line with the results of our study. The mentioned variables are important factors contributing to both the development and prevention of osteoporosis. One of the factors that has a pivotal role in preventing osteoporosis are self-care behaviors. The findings of the present study showed that in all domains of self-care, women with osteoporosis had lower scores.

Self-care includes different fields in this direction. Nikpour et al. reported a significant relationship between the consumption pattern of foods rich in vitamin D and calcium and the development of osteoporosis. This cross-sectional study was conducted on 500 women referring to bone density measurement centers of Iran University of Medical Sciences. It has also been argued that sports activities lead to better absorption of calcium in the digestive system and that the presence of sufficient amounts of calcium reduces the production of parathormone hormone, hence reducing the bone resorption of calcium, which affects the positive changes in bone mass [23]. Also in line with the results of our research, the study of Fallahi et al. was conducted using a qualitative method with a content analysis approach. Fifteen women referred to bone density measurement centers in Sanandaj city participated in this research based on purpose-based sampling [11]. They pointed out that women with osteoporosis have problems with self-care. Factors such as disability and hope against the disease, the mutual roles of the doctor, and the role of the family and organizations and executive centers are effective in the self-care status of women with osteoporosis.

Meanwhile, health literacy is one of the important components of self-care. According to our results, all healthy women in our study (100%) had sufficient health literacy, whereas 52.8% of osteoporotic women had insufficient health literacy. In addition, there was a statistically significant difference between the two groups in terms of their health literacy levels. Health literacy is one of the main factors affecting women’s health [21], and the results of previous studies also show the same thing. Schillinger in a cross-sectional observational study on 408 English and Spanish-speaking patients who were over 30 years old and had type 2 diabetes showed that people with insufficient health literacy have weaker self-care skills, poor reporting of their health, lower preventive practices, higher treatment costs, more frequent visits to doctors, long-term hospitalization, poor communication with the medical staff [15]. Also, in a study conducted by Lisa et al. on 332 patients in a preoperative clinic, it was shown that weaker management in controlling chronic diseases, insufficient knowledge about their treatment conditions, and weaker participation in making decisions related to their treatment [24]. Although these studies were conducted on people with different health problems, they all finally confirm that insufficient health literacy is associated with poor self-care behaviors.

The results of the present study showed that there is a significant relationship between self-care behaviors and the level of health literacy in total osteoporotic and healthy women. In line with the results of this study, Chen et al. also reported a positive relationship between health literacy and adherence to self-care practices in patients with chronic kidney disease (CKD) [25]. The literature abounds with studies consistent with the present study, and most of these studies have been done in clinical settings. Shin and Lee, for example, found that increased health literacy results in the promotion of self-care behaviors in diabetic patients [26]. In addition, Wang et al.’s study on patients with CKD concluded that health literacy plays an important role in self-care behaviors [27].

Barati et al. found a positive and significant correlation between various domains of health literacy and self-care behaviors in patients with high blood pressure. That is, the higher the level of health literacy of patients, the higher their level of self-care. Therefore, insufficient or lack of health literacy prevents people from having a correct understanding of their illness and the ways to deal with it[28]. Health literacy is thus a vital indicator in health care outcomes and costs, and if left ignored, longer use of medical services will be unavoidable [29].

Tira et al. (2014) considered self-care as one of the critical factors for better management and control of diseases, and they admitted that self-care behaviors are closely tied to patients’ health literacy levels [30]. Therefore, insufficient health literacy is regarded as a serious obstacle to disease management and can negatively affect patients’ participation in self-care practices [31].

Osteoporotic patients need sufficient knowledge and a proper attitude toward self-care for them to successfully control their disease. Noureldin et al. found that patients with sufficient health literacy have better compliance with their medication regimen compared with insufficiently health-literate patients, confirming that health literacy is an influential factor in sustainable drug interventions [32].

According to the results of the current study, there was a significant relationship between self-care and health literacy, and this relationship can be extended to the domains of self-care, with each of these domains having a significant relationship with the level of health literacy. The importance and necessity of paying attention to health literacy in therapeutic interventions carried out to promote self-care in patients was well established in this study. In fact, it is imperative to pay attention to the level of health literacy and self-care in patients since these are important factors influencing treatment adherence and compliance with medical advice. Our results also showed a strong significant relationship between self-care behaviors and the level of health literacy in older women with osteoporosis, but this relationship was not observed in healthy aged women, which can be attributed to the high level of self-care and health literacy in all women of this subgroup and the homogeneity of the group in terms of these components.

This study has some strengths including a substantial number of subjects, the translation of the questionnaire using appropriate tools, the inclusion of a real healthy population (at least from a bone health point of view), and selection high-risk population i.e. older women as the study population. The main limitation of this study was a large number of items in the questionnaires, which could bore the respondents and make them tired. To alleviate this problem, the participants were asked to complete the questionnaires in a quiet environment in the center and with intervals between the questionnaires. It should be noted that the present investigation was of an exploratory nature, lacking any preliminary data, and the sample size was determined based on nutrition scores in healthy and osteoporotic-aged women. Subsequent to this pilot exploratory study, it is imperative to substantiate the findings in a cohort study. The results of this study cannot be generalized to aged women with secondary osteoporosis. Also, this study investigated the status of self-care and health literacy in healthy and osteoporotic older women in a particular city in Iran, so its results cannot be generalized to older women with other diseases or living in other cities. Therefore, future studies are recommended to study this relationship in patients with other diseases and living in other cities.

## Conclusions

The results of the present study showed that self-care behavior and health literacy play important preventive roles in older women’s osteoporosis. Based on the results, the health literacy of older women has a significant effect on their self-care. In other words, the higher the health literacy of older women, the higher their self-care practices. Therefore, it should be stated that the issue of self-care is of paramount importance not only in aged women with osteoporosis but also in those who are healthy or at risk of developing it. Therefore, empowering women concerning health literacy is an important factor in promoting self-care behaviors and ultimately the health of this population. Health officials and policymakers are thus strongly recommended to take into account health literacy as one of the most important tools to improve self-care. The results of the present study can provide insights into the formulation, design, and implementation of action plans for all aged populations, especially older women.

### Electronic supplementary material

Below is the link to the electronic supplementary material.

**Additional File 1**: Menopausal women self-care questionnaire

## Data Availability

The datasets used and analyzed during the current study available from the corresponding author on reasonable request.
