# Supplementary material for: The self-care behaviors and health literacy can play important preventive roles in older female osteoporosis
Source: BMC Womens Health. 2023 Aug 11;23:429. doi: 10.1186/s12905-023-02546-2 (PMC10422789; doi:10.1186/s12905-023-02546-2)
Supplement: Supplementary file 1 — Additional File 1: Menopausal women self-care questionnaire [file 12905_2023_2546_MOESM1_ESM.docx]

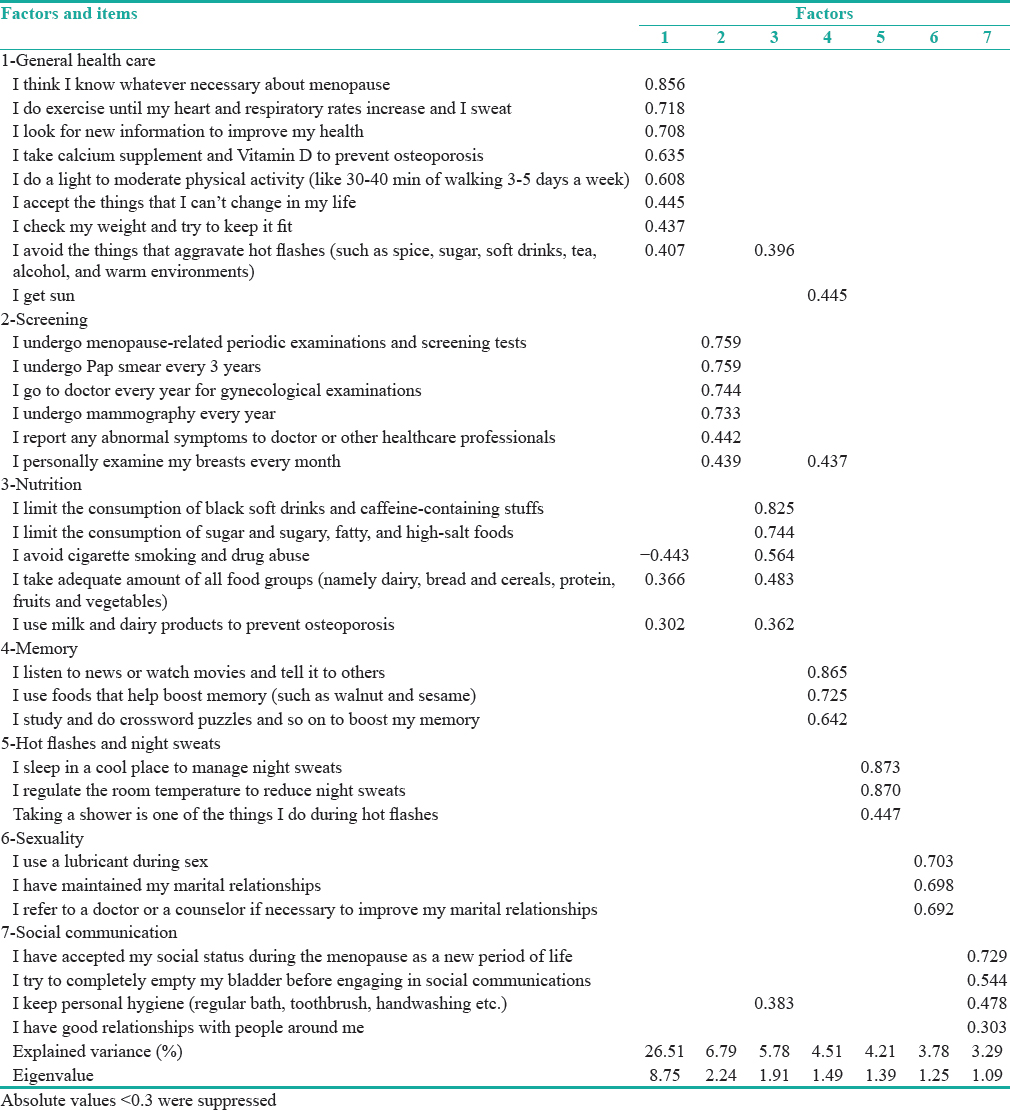


"Questionnaires used"

**Menopausal women self-care questionnaire**

| Factors and items | Never  (1) | rarerarely (2) | sometimes  (3) | Usually  (4) | Always  (5) |
| --- | --- | --- | --- | --- | --- |
| General health care |  |  |  |  |  |
| I think I know whatever necessary about menopause |  |  |  |  |  |
| I do exercise until my heart and respiratory rates increase and I sweat |  |  |  |  |  |
| I look for new information to improve my health |  |  |  |  |  |
| I take calcium supplement and Vitamin D to prevent osteoporosis |  |  |  |  |  |
| I do a light to moderate physical activity (like 30-40 min of walking 3-5 days a week) |  |  |  |  |  |
| I accept the things that I can’t change in my life |  |  |  |  |  |
| I check my weight and try to keep it fit |  |  |  |  |  |
| I avoid the things that aggravate hot flashes (such as spice, sugar, soft drinks, tea, alcohol, and warm environments) |  |  |  |  |  |
| I get sun |  |  |  |  |  |
| Screening |  |  |  |  |  |
| I undergo menopause‑related periodic examinations and screening tests |  |  |  |  |  |
| I undergo Pap smear every 3 years |  |  |  |  |  |
| I go to doctor every year for gynecological examinations |  |  |  |  |  |
| I undergo mammography every year |  |  |  |  |  |
| I report any abnormal symptoms to doctor or other healthcare professionals |  |  |  |  |  |
| I personally examine my breasts every month |  |  |  |  |  |
| Nutrition |  |  |  |  |  |
| I limit the consumption of black soft drinks and caffeine‑containing stuffs |  |  |  |  |  |
| I limit the consumption of sugar and sugary, fatty, and high‑salt foods |  |  |  |  |  |
| I avoid cigarette smoking and drug abuse |  |  |  |  |  |
| I take adequate amount of all food groups (namely dairy, bread and cereals, protein, fruits and vegetables) |  |  |  |  |  |
| I use milk and dairy products to prevent osteoporosis |  |  |  |  |  |
| Memory |  |  |  |  |  |
| I listen to news or watch movies and tell it to others |  |  |  |  |  |
| I use foods that help boost memory (such as walnut and sesame) |  |  |  |  |  |
| I study and do crossword puzzles and so on to boost my memory |  |  |  |  |  |
| Hot flashes and night sweats |  |  |  |  |  |
| I sleep in a cool place to manage night sweats |  |  |  |  |  |
| I regulate the room temperature to reduce night sweats |  |  |  |  |  |
| Taking a shower is one of the things I do during hot flashes |  |  |  |  |  |
| Sexuality |  |  |  |  |  |
| I use a lubricant during sex |  |  |  |  |  |
| I have maintained my marital relationships |  |  |  |  |  |
| I refer to a doctor or a counselor if necessary to improve my marital relationships |  |  |  |  |  |
| Social communication |  |  |  |  |  |
| I have accepted my social status during the menopause as a new period of life |  |  |  |  |  |
| I try to completely empty my bladder before engaging in social communications |  |  |  |  |  |
| I keep personal hygiene (regular bath, toothbrush, handwashing etc.) |  |  |  |  |  |
| I have good relationships with people around me |  |  |  |  |  |

**European Health Literacy (HLS-EU)-Q16**

| **HLS-EU-Q16 Item** | **Difficult to Say/Not** | | **Very Difficult %** | **Fairly Difficult %** | **Fairly Easy %** | **Very Easy %** |
| --- | --- | --- | --- | --- | --- | --- |
| 1. Find information on treatments of illnesses that concern you? | |  |  |  |  |  |
| 2. Find out where to get professional help when you are ill? | |  |  |  |  |  |
| 3. Understand what your doctor says to you? | |  |  |  |  |  |
| 4. Understand your doctor’s or pharmacist’s instruction on how to take a prescribed medicine? | |  |  |  |  |  |
| 5. Judge when you may need to get a second opinion from another doctor? | |  |  |  |  |  |
| 6. Use information the doctor gives you to make decisions about your illness? | |  |  |  |  |  |
| 7. Follow instructions from your doctor or pharmacist? | |  |  |  |  |  |
| 8. Find information on how to manage mental health problems like stress or depression? | |  |  |  |  |  |
| 9. Understand health warnings about behaviour such as smoking, low physical activity and drinking too much? | |  |  |  |  |  |
| 10. Understand why you need health screenings? | |  |  |  |  |  |
| 11. Judge if the information on health risks in the media is reliable? | |  |  |  |  |  |
| 12. Decide how you can protect yourself from illness based on information in the media? | |  |  |  |  |  |
| 13. Find out about activities that are good for your mental well-being? | |  |  |  |  |  |
| 14. Understand advice on health from family members or friends? | |  |  |  |  |  |
| 15. Understand information in the media on how to get healthier? | |  |  |  |  |  |
| 16. Judge which everyday behaviour is related to your health? | |  |  |  |  |  |
